# Favorable Outcomes in a Rare Case of Chiari Malformation Type III: A Clinical Report

**DOI:** 10.7759/cureus.84482

**Published:** 2025-05-20

**Authors:** Renz Marion M Alemania, Maria Estrella G Ibe-Ilustre

**Affiliations:** 1 Department of Neurology, Jose R. Reyes Memorial Medical Center, Manila City, PHL; 2 Department of Pediatrics, Jose R. Reyes Memorial Medical Center, Manila City, PHL

**Keywords:** chiari malformation type iii, encephalocoele, hydrocephalus, posterior cranial fossa, surgery

## Abstract

This report details the case of a 1-year and 11-month-old Filipino female patient who presented with a persistent occipital mass that was soft, non-tender, mobile, and lit through upon transillumination. A neurological test revealed a global developmental delay. Neuroimaging revealed a bony defect at the posterior cranial fossa with herniation of the occipital lobe, cerebrospinal fluid, and the meninges, consistent with Chiari malformation type III, a rare and severe form accounting for less than 1% of Chiari malformations. While this condition typically carries a poor prognosis, the patient showed a favorable outcome following neurosurgical intervention.

We present this case to share our clinical experience with this exceedingly rare condition and highlight its potential for positive post-surgical outcomes.

## Introduction

Chiari malformations (CM) are congenital anomalies characterized by structural defects of the posterior cranial fossa, often accompanied by herniation of cerebellar tissue through the foramen magnum into the upper spinal canal [1]. These malformations are frequently associated with other neurological anomalies such as syringomyelia, hydrocephalus, and scoliosis. The pathophysiology of syrinx formation in CM is thought to involve the obstruction of cerebrospinal fluid (CSF) flow at the foramen magnum, both intramedullary and extramedullary [2]. Scoliosis in CM has been hypothesized to result from increased pressure within the syrinx, disrupting the function of postural muscles and leading to spinal curvature [1,2].

CM is classified into various types based on severity and morphological features. The most common is type I, which presents with downward displacement of the cerebellar tonsils and is estimated to occur in 0.8-3.7% of children [3]. Type II involves herniation of the cerebellar vermis and tonsils into the foramen magnum and is often associated with myelomeningocele. Type III is characterized by herniation of posterior fossa contents into a cervical or occipital encephalocele, with a prevalence of approximately 0.64%, or about two cases in every 312 CM cases reported [4]. Type IV involves cerebellar hypoplasia and is the least common [2].

Common neurological associations vary by type. Type I often coexists with tethered cord, syringomyelia, and scoliosis; type II with tethered cord and scoliosis; type III frequently with encephalocele and hydrocephalus; and type IV with cerebellar hypoplasia [1,5,6]. Additionally, CM may be associated with cardiovascular anomalies, such as atrial septal defects, patent ductus arteriosus, and ventricular septal defects, as documented by Horn et al. [1].

CM type III is exceptionally rare, with only around 34 cases described in the literature. It typically presents with herniation of posterior fossa structures associated with hydrocephalus, syringomyelia, and tethered cord syndrome. Magnetic resonance imaging (MRI) remains the imaging modality of choice for diagnosis, with prenatal ultrasonography and congenital anomaly scans capable of detecting posterior fossa herniation and hydrocephalus prenatally [4,7].

Surgical management involves excision of the encephalocoele, aiming to preserve neurological function and prevent compression due to hydrocephalus [8,9]. Although literature suggests a generally poor prognosis for CM type III, recent reports have demonstrated favorable outcomes post-surgery [8,10]. Despite surgical intervention, many patients continue to experience varying degrees of neurological deficits, including intellectual disability and motor delays, and even mortality. This case report highlights a patient with CM type III who achieved a positive neurologic outcome following surgical treatment.

## Case presentation

We report a case of a 1-year and 11-month-old Filipino female from San Pablo City, Laguna, Philippines, who consulted our institution with a persistent occipital mass. The mass was first noted during a third-trimester ultrasound. On physical examination, the mass was soft, non-tender, movable, and demonstrated light through a transillumination test. Neurological assessment revealed global developmental delay, with delays across all domains of function by approximately six to eight months compared to age-appropriate milestones.

**Figure 1:**
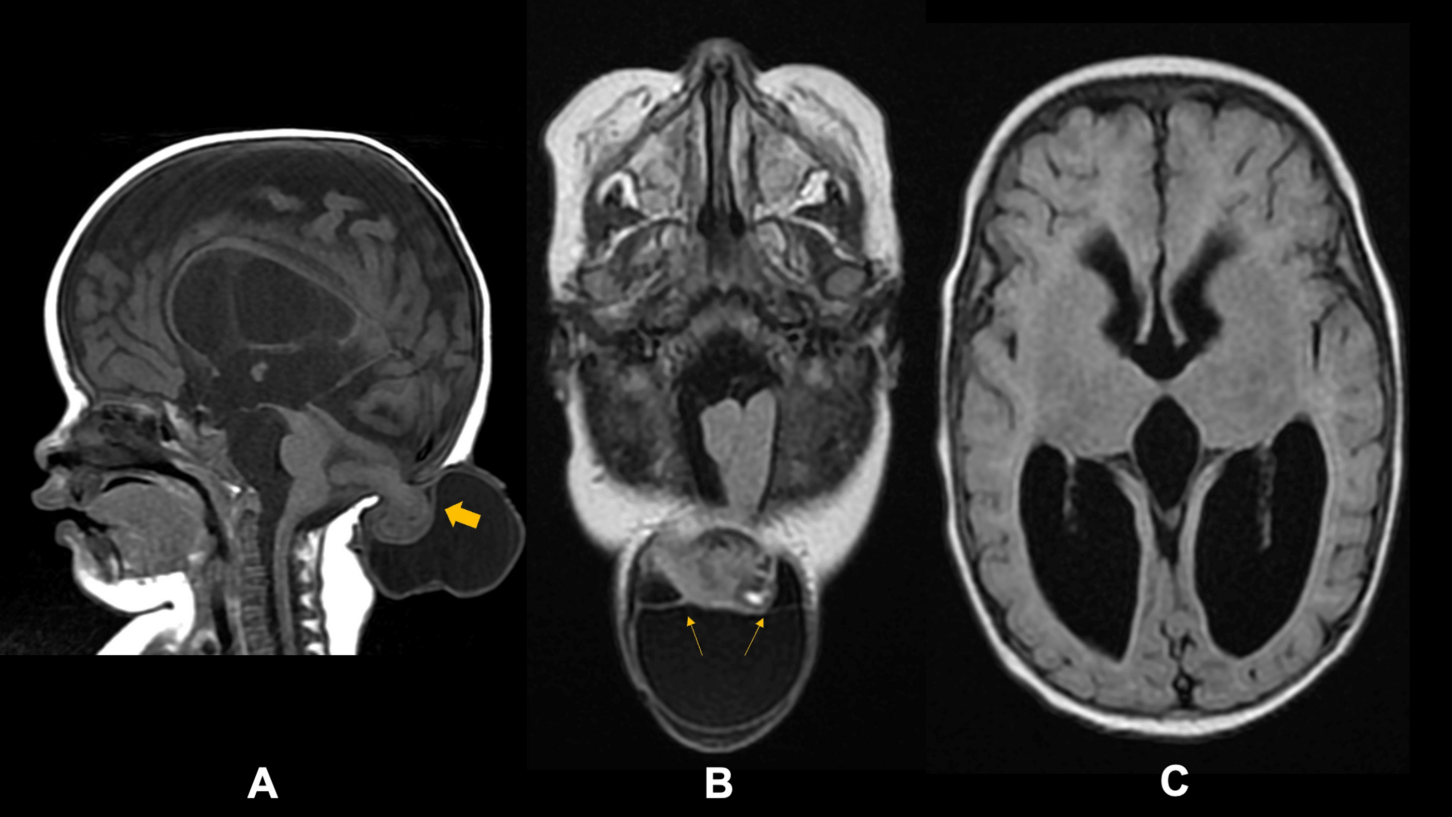
Multiplanar and multisequential MR scans of the brain were obtained using the 1.5 Tesla GE Signa Voyager Scanner (GE Healthcare, Chicago, Illinois, US) Cranial MR imaging showed extracranial herniation of SF and some of the posterior fossa structures composed of the right cerebellar hemisphere (dark arrow) and portion of the vermis (A) measuring approximately 2.5 x 2.5 x 2.1 cm (CCWAP; 4.4 x 4.5 x 5.2 cm including the SF component), through a midline low occipital bone defect (diameter of 1.1 cm). There is associated herniation of the meninges with some septations (thin arrow) and folding of the dura (B). There is a subsequent posterior traction effect on the cerebellar vermis and brainstem, with elongation of the fourth ventricle and aqueduct of Sylvius. There is consequent dilatation of the third and lateral ventricles (C). There is thinning of the corpus callosum and compression of the cerebral hemispheres. *Abbreviations: *MRI: Magnetic resonance imaging; SF: spinal fluid; SWI: susceptibility-weighted imaging; GRE: gradient-recall echo; FLAIR: fluid-attenuated inversion recovery *Legend: *A: T1 sagittal; B and C: T2 FLAIR axial

The patient was managed as a case of occipital meningoencephalocele with secondary hydrocephalus, consistent with Chiari malformation type III. To further evaluate, cranial magnetic resonance imaging (MRI) with angiography and venography was performed (Figure 1). Neuroimaging revealed extracranial herniation of posterior fossa structures through a midline low occipital bony defect, along with herniated meninges exhibiting septations and folding of the dura. Additionally, there was elongation of the fourth ventricle and aqueduct of Sylvius, with dilatation of the third and lateral ventricles. The time-of-flight angiogram and venogram were unremarkable.

A neurosurgical consult was done. The patient underwent repair of encephalocoele with ventriculoperitoneal shunt (VPS) insertion. The right VPS was inserted with high opening pressure, and the pedunculated encephalocoele was repaired. Postoperatively, the patient tolerated the procedure well, demonstrating good activity, good cry, and adequate feeding. The postoperative site was dry, with no signs of infection. There was no indication of increased intracranial pressure on routine examination.

Following surgery, the patient was observed for any untoward postoperative complications and was referred to a neurodevelopmental specialist and rehabilitation medicine for management of the developmental delay. She was discharged in good condition with no febrile episodes or any deterioration of sensorium. The patient was given a schedule for follow-up and monitoring post-discharge.

## Discussion

Chiari malformation type III is an exceptionally rare congenital condition, accounting for approximately 0.64% of Chiari malformation cases, and is associated with a generally poor prognosis [1]. It is characterized by an occipital or high cervical encephalocele containing dysplastic neural tissue, along with osseous defects. Unlike other Chiari types, CM type III often presents with herniation of posterior fossa contents and hydrocephalus, which can complicate management [3].

The exact pathogenesis of CM type III remains unclear; however, several hypotheses have been proposed. These include a primary abnormality in mesodermal development, leading to defective formation of the occipital bone and posterior fossa [4], and an abnormal escape of CSF through an open neural tube defect during fetal development, hindering normal expansion of the embryonic ventricles, which results in a small skull and disorganized posterior fossa [2,4]. Some theories suggest that a lack of proper distension of the embryonic ventricular system causes posterior fossa hypoplasia, leading to brain tissue displacement and herniation [2].

The clinical presentation of CM III varies depending on the amount and type of neural tissue within the encephalocele, as well as coexisting anomalies [1-3]. Symptoms can range from asymptomatic to severe neurologic deficits, including cerebellar signs such as titubation and downbeat nystagmus, sensory loss, weakness, ataxia, respiratory insufficiency or failure, abnormal muscle tone, dysphagia with aspiration risk, and inspiratory stridor [1,3,4].

Histopathologically, the encephalocele typically contains gliosis, necrosed neural tissue, meningeal inflammation, fibrosis, cerebral and cerebellar tissues, ventricles with choroid plexuses, heterotopic gray matter, and reactive astrocytosis [4]. Prenatal ultrasonography allows the early detection of encephaloceles, usually around 18 weeks of gestation, with MRI providing detailed characterization of brain and posterior fossa anomalies, aiding in diagnosis, prognosis, and birth planning [2,4].

CM type III is associated with various anomalies, including hydrocephalus, syringomyelia, hematomas within herniated cerebellar tissue, congenital heart disease, scoliosis, tectal beaking, and holocord syrinx [1,3,4]. Some patients also exhibit craniofacial anomalies such as microcephaly, hypertelorism, syndactyly, clinodactyly, and polydactyly [1].

Without surgical treatment, CM Type III often results in death due to progressive herniation and associated complications [2,3]. Surgical management involves primary closure of the encephalocele, resection of nonfunctional tissue when appropriate, dural repair to restore CSF flow, and measures to prevent tethering. The procedure's goal is to improve neurological outcomes and quality of life, although prognosis remains guarded due to the severity of associated anomalies [3,4].

## Conclusions

Chiari malformation type III is an exceedingly rare congenital malformation. It carries a poor prognosis, but can be life-compatible with appropriate management. Timely diagnosis allows for strategic planning to prevent complications, including herniation, tethering, impaired cerebrospinal fluid flow, and neurological deterioration, ultimately improving patient outcomes. This case contributes to the limited body of literature by highlighting a favorable outcome in a resource-limited setting, emphasizing that multidisciplinary management and early intervention are essential for optimizing prognosis and preserving neurological function, even where advanced resources may be scarce. Future reports are needed to determine the possible factors contributing to positive outcomes in these rare cases and to optimize treatment strategies for diverse settings.
